# CLAG-M with dose-escalated mitoxantrone for adults with acute myeloid leukemia

**DOI:** 10.18632/oncotarget.26383

**Published:** 2018-11-27

**Authors:** Anna B. Halpern, Roland B. Walter

**Affiliations:** Roland B. Walter: Department of Medicine, Division of Hematology, University of Washington, Seattle, WA, USA; Clinical Research Division, Fred Hutchinson Cancer Research Center/University of Washington, Seattle, WA, USA; Department of Epidemiology, University of Washington, Seattle, WA, USA

**Keywords:** acute myeloid leukemia, adult patients, CLAG-M, intensive chemotherapy, phase 1/2 trial

Intensive multi-agent chemotherapy is an important cornerstone of curative-intent treatment for acute myeloid leukemia (AML). This is unlikely to change soon despite recent approval of several new targeted drugs (e.g. gemtuzumab ozogamicin, midostaurin) which may be used best as add-on to, rather than replacement of, conventional chemotherapeutics. Numerous regimens have been developed for AML therapy, including standard-dose cytarabine with an anthracycline (e.g. “7+3”), the combination of mitoxantrone, etoposide and cytarabine (“MEC”), or the combination of fludarabine, cytarabine, granulocyte colony stimulating factor (G-CSF) and idarubicin (“FLAG-Ida”), among others. Since established regimens are ineffective in many patients, however, there is ongoing interest in new or refined chemotherapy backbones for people with newly-diagnosed as well as relapsed/refractory AML.

In 2008, Polish investigators reported cladribine (5 mg/m^2^ on days 1-5), cytarabine (2,000 mg/m^2^ on days 1-5), G-CSF, and mitoxantrone (10 mg/m^2^ on days 1-3; “CLAG-M”) in adults with primary refractory AML or early relapsed disease had acceptable tolerability and encouraging activity – among 118 treated people, a complete remission (CR) rate of 58% was obtained [1]. This regimen has not yet been directly compared to other regimens in a controlled fashion but retrospective analyses of consecutive patients treated at a single institution indicated CLAG (i.e. CLAG-M without mitoxantrone) may be superior to MEC in the relapsed/refractory disease setting [2].

Because randomized trials demonstrated dose-dependent anti-leukemic efficacy of anthracyclines during induction chemotherapy [3-5], we recently conducted a single-center phase 1/2 trial (ClinicalTrials.gov: NCT02044796) testing CLAG-M with escalated doses of mitoxantrone in separate cohorts of fit adults ≥18 years with either newly-diagnosed or relapsed/refractory AML and other high-grade myeloid neoplasms (≥10% myeloblasts in blood and/or marrow) [6, 7]. In both cohorts, medical fitness was defined by a treatment-related mortality (TRM) score of ≤6.9, a score that corresponds to a ≤6.9% probability of 4-week mortality [8]. Patients were also required to have a left ventricular ejection fraction ≥45%, a serum creatinine ≤2.0 mg/dL, a total bilirubin ≤2.5 times the upper limit of normal, no uncontrolled infection, and expected survival of >1 year absent AML. In phase 1, groups of 6-12 patients received 12, 14, 16, or 18 mg/m^2^ of mitoxantrone on days 1-3 to determine the recommended phase 2 dose (RP2D), which was then used for subsequent patients.

In the newly-diagnosed arm, we enrolled 121 patients with a median age of 60 (range: 21-81) years [6]. Because all 4 mitoxantrone doses were well-tolerated, 18 mg/m^2^ was declared the RP2D in this arm. 74/94 (79%) patients treated at the RP2D achieved a CR, 67 of whom had no measurable residual disease (MRD) by multiparameter flow cytometry for an overall MRD^neg^ CR rate of 71%. In addition, 7 patients achieved a CR with incomplete hematologic recovery (CRi; 7%, 5 MRD^neg^) for a CR/CRi rate of 81/94 (86%). 4-week mortality was 2%. After adjustment for various prognostic factors, age was not associated with response. The MRD^neg^ CR and CR/CRi rates with CLAG-M compared favorably to 100 matched controls treated with 7+3 at our center and 245 matched patients treated with 7+3 on a contemporary cooperative group trial; toxicity rates and duration of cytopenias appeared similar.

In the relapsed/refractory arm, we enrolled 60 patients, median age 61 (range: 33-76) years, with primary refractory (*n* = 25) or relapsed disease (*n* = 35) who received a median of 2 (range: 1-6) prior therapies [7]. Because of 2 dose-limiting toxicities (encephalopathy and cardiogenic shock) at the highest tested dose, mitoxantrone at 16 mg/m^2^ was defined as the RP2D in this arm. Eleven (28%) and 13 (32%) patients treated at that dose achieved a CR or CRi for a CR/CRi rate of 60% (19/24 [80%] without MRD). 4- and 8-week mortality was 5% at both timepoints. Comparisons between patients with primary refractory *vs*. relapsed disease revealed no difference in tolerability or rates of subsequent hematopoietic cell transplantation. After adjustment, CLAG-M with mitoxantrone at 16 mg/m^2^ was associated with statistically significantly longer overall survival and non-significantly longer relapse-free survival compared to 30 similar patients treated at our institution with CLAG-M with regular-dose mitoxantrone, while remission rates were not different. After controlling for prognostic factors, CR/CRi rates were higher, and survival longer, in people treated with CLAG-M at 16 mg/m^2^ compared to 36 matched patients treated with decitabine-primed MEC (d/MEC [9]) but not 56 patients treated with G-CSF/clofarabine/cytarabine (GCLAC [10]).

Overall, the results from our institutional phase 1/2 trial indicate CLAG-M with mitoxantrone up to 18 mg/m^2^ (newly-diagnosed disease) or 16 mg/m^2^ (relapsed/refractory disease) are safe and relatively well-tolerated in fit younger and older adults with AML and other high-grade myeloid neoplasms; whether this RP2D difference reflects a greater vulnerability of patients with relapsed/refractory disease to higher doses of mitoxantrone or represents a chance finding due to the small patient numbers is unknown. Escalating the mitoxantrone dose may provide additional anti-leukemia efficacy over the dose conventionally used with CLAG-M, although non-randomized comparisons and small cohort sizes limit the conclusions we can draw from our analyses.

Our study adds to the evidence supporting the use of CLAG-M for intensive AML re-induction therapy, perhaps particularly with higher mitoxantrone doses. Perhaps more importantly, our study points to a role of this regimen in the upfront setting where it is currently not commonly used. Demonstration of a higher rate of MRD^neg^ remissions than with 7+3 – most achieved with the first cycle of therapy – makes CLAG-M an attractive regimen for initial therapy of AML although randomized comparisons are needed to validate this finding. Because of the general tolerability of CLAG-M with escalated doses of mitoxantrone, institutional efforts are ongoing to test add-on of sorafenib (NCT02728050) or gemtuzumab ozogamicin (NCT03531918) in fit younger and older adults with newly-diagnosed AML. In parallel, this regimen is currently in medically less-than-fit adults with newly-diagnosed AML.
